# Patient Derived Colonoids as Drug Testing Platforms–Critical Importance of Oxygen Concentration

**DOI:** 10.3389/fphar.2021.679741

**Published:** 2021-05-13

**Authors:** Helene Kolstad Skovdahl, Shreya Gopalakrishnan, Tarjei Dahl Svendsen, Atle van Beelen Granlund, Ingunn Bakke, Zekarias G. Ginbot, Silje Thorsvik, Arnar Flatberg, Bjørnar Sporsheim, Jenny Ostrop, Tom Eirik Mollnes, Arne Kristian Sandvik, Torunn Bruland

**Affiliations:** ^1^ Department of Clinical and Molecular Medicine (IKOM), Faculty of Medicine and Health Sciences, NTNU- Norwegian University of Science and Technology, Trondheim, Norway; ^2^ Centre of Molecular Inflammation Research (CEMIR), Faculty of Medicine and Health Sciences, NTNU- Norwegian University of Science and Technology, Trondheim, Norway; ^3^ Clinic of Laboratory Medicine, St. Olav's University Hospital, Trondheim, Norway; ^4^ Department of Gastroenterology and Hepatology, Clinic of Medicine, St. Olav's University Hospital, Trondheim, Norway; ^5^ Central Administration, St Olavs Hospital, The University Hospital in Trondheim, Trondheim, Norway; ^6^ Research Laboratory, Nordland Hospital, Bodø, Norway; ^7^ K.G. Jebsen Thrombosis Research and Expertise Centre, Institute of Clinical Medicine, University of Tromsø, Tromsø, Norway; ^8^ Department of Immunology, Oslo University Hospital and University of Oslo, Oslo, Norway; ^9^ Clinic of Medicine, St. Olav's University Hospital, Trondheim, Norway

**Keywords:** inflammatory bowel diseases, epithelium, organoids, oxygen, cytokines, tumor necrosis factor, interleukin 17

## Abstract

Treatment of inflammatory bowel disease (IBD) is challenging, with a series of available drugs each helping only a fraction of patients. Patients may face time-consuming drug trials while the disease is active, thus there is an unmet need for biomarkers and assays to predict drug effect. It is well known that the intestinal epithelium is an important factor in disease pathogenesis, exhibiting physical, biochemical and immunologic driven barrier dysfunctions. One promising test system to study effects of existing or emerging IBD treatments targeting intestinal epithelial cells (IECs) is intestinal organoids (“mini-guts”). However, the fact that healthy intestinal epithelium is in a physiologically hypoxic state has largely been neglected, and studies with intestinal organoids are mainly performed at oxygen concentration of 20%. We hypothesized that lowering the incubator oxygen level from 20% to 2% would recapitulate better the *in vivo* physiological environment of colonic epithelial cells and enhance the translational value of intestinal organoids as a drug testing platform. In the present study we examine the effects of the key IBD cytokines and drug targets TNF/IL17 on human colonic organoids (colonoids) under atmospheric (20%) or reduced (2%) O_2_. We show that colonoids derived from both healthy controls and IBD-patients are viable and responsive to IBD-relevant cytokines at 2% oxygen. Because chemokine release is one of the important immunoregulatory traits of the epithelium that may be fine-tuned by IBD-drugs, we also examined chemokine expression and release at different oxygen concentrations. We show that chemokine responses to TNF/IL17 in organoids display similarities to inflamed epithelium in IBD-patients. However, inflammation-associated genes induced by TNF/IL17 were attenuated at low oxygen concentration. We detected substantial oxygen-dependent differences in gene expression in untreated as well as TNF/IL17 treated colonoids in all donors. Further, for some of the IBD-relevant cytokines differences between colonoids from healthy controls and IBD patients were more pronounced in 2% O_2_ than 20% O_2_. Our results strongly indicate that an oxygen concentration similar to the *in vivo* epithelial cell environment is of essence in experimental pharmacology.

## Introduction

Ulcerative colitis (UC) and Crohn’s disease (CD), collectively termed inflammatory bowel disease (IBD), are prevalent and chronic conditions with an incompletely understood pathobiology involving the immune system, intestinal epithelium and microbiota. The intestinal epithelial cell (IEC) monolayer separates gut microbiota from lamina propria immune cells and influences signaling between microbes and the immune system, shaping the homeostatic environment of the healthy gut (Kaser et al., 2010). These processes involve *e.g.* secretion of mucus, antimicrobial peptides, IgA and an array of cytokines, responding to microbes through pattern recognition receptors (PRRs) (Allaire et al., 2018).

Although there is no cure for IBD, drugs modify disease course. Basic therapeutics are glucocorticoids and 5-aminosalicylic acid, and insight into disease mechanisms has led to biological drugs aimed at cytokines such as *e.g.* anti-TNF and anti-IL12/23p40, at α4β7 lymphocyte homing mechanisms, and lately drugs inhibiting JAK-STAT pathways (Chang and Hudesman, 2020). Other promising drugs are under development but not yet approved. A central problem is that no treatment helps all patients, and little predictive information can be derived from disease traits of the individual patient. For instance, anti-TNF biologicals are the primary drugs after failure of basic treatment, but 30% of patients are primary nonresponders and 40% of those with an initial effect lose response (Ben-Horin and Chowers, 2011; Yanai and Hanauer, 2011).

Patients may face time-consuming drug trials while the disease is active, thus there is an unmet need for biomarkers and assays to predict drug effect. One promising test system is intestinal organoids, first developed for stem cell research, disease modelling and regenerative medicine (Sato and Clevers, 2013). In IBD, organoids may be used as patient-specific *in vitro* assays to predict the clinical response to drugs targeting the IEC. This would be an important tool for personalized medicine, and may reveal new and more easily applicable biomarkers for prediction of treatment response.

Colonic epithelial organoids (colonoids) can be derived from pinch biopsies with a high success rate. The process is labour-intensive and cannot be done for all IBD patients. However, cryogenic preservation of biopsies has made it possible to generate colonoids at a later point of time for patients with difficult disease (Tsai et al., 2018). A potential problem has been seen in colonoids looking at IEC characteristics in UC patients *in vitro*, where results diverge. Some studies find persisting abnormalities possibly due to genetic or epigenetic factors (Dotti et al., 2017), while in others UC characteristics disappear (Arnauts et al., 2020). The central question thus becomes how IEC pathobiology and drug responses can be studied in colonoids retaining UC characteristics.

The low level of oxygen in the colonic mucosa might be one important factor in this regard, with oxygen concentration decreasing from approximately 3% at the crypt base to <1% at the surface (Litvak et al., 2018; Keeley and Mann, 2019). This has largely been neglected, and studies on colonoids have been performed using a standard cell culture oxygen concentration of 20%. Thus, the aim of the present study was to examine the inflammatory responses in colonoids from patients with UC, as compared to healthy individuals, and how an oxygen concentration of 2% retains colon IEC characteristics and changes responses to two relevant and important IBD related cytokines, TNF and IL17.

## Methods

### Ethics Approval

The study was approved by the Central Norway Regional Committee for Medical and Health Research Ethics (Reference numbers 5.2007.910 and 2013/212/REKmidt). Informed written consent was obtained from all subjects included in the study.

### Patient Material

Patients were included in the study after referral to the Department of Gastroenterology and Hepatology for colonoscopy. Patients either received or had a prior diagnosis of UC or CD, or were included as controls after clinical assessment and a normal colonoscopy. They were categorized as healthy controls, or UC or CD with inactive disease or ongoing inflammation, based on clinical, endoscopic and histological evaluation. Colonic pinch biopsies were collected and preserved in formalin or snap frozen in liquid nitrogen. Histopathology was evaluated in hematoxylin and eosin stained sections by an expert pathologist as previously described (Granlund et al., 2013). Inflamed samples were taken from maximally inflamed area with intact epithelium. Un-inflamed samples, and samples from healthy controls, were taken from the hepatic flexure. A sample was only included in the final analysis if there was full concordance between endoscopic and histopathological assessment of inflammatory status. Colonic biopsies from 29 subjects (active IBD (*n* = 12), uninflamed IBD (*n* = 11) and healthy controls (*n* = 6)) underwent laser capture microdissection of the epithelial monolayer, as described (Ostvik et al., 2020). Biopsies from uninflamed mucosa at the hepatic flexure were taken from an additional six patients (HC = 3, UC = 3) to create colonoids for functional assays (Supplementary File SF1).

### Human Colonoid 3D Culture and Treatment

Human colonoids were established from epithelial crypts collected from colonic pinch biopsies using an optimized protocol from (Mahe et al., 2015) based on (Jung et al., 2011). Epithelial crypts were isolated from biopsies and resuspended in basement membrane matrix (#734–1101, Matrigel^®^ Growth Factor Reduced (GFR) Basement Membrane Matrix, phenol red-free, Corning^®^, New York City, NY, United States), and crypt suspension was added to pre-warmed plates with 50µl crypt suspension per well in 24-well plates, and 10µl crypt suspension per well in 96-well plates. Growth medium (500µl per well in 24-well plates, 100µl in 96-well plates) contained 50% Wnt-3A conditioned medium (#CRL-2647, RRID:CVCL_0635, ATCC, Manassas, VA, United States), 30% Advanced DMEM/F12 (#12634028, Thermo Fischer Scientific, Bremen, Germany), and 20% R-spondin conditioned medium (HA-R-Spondin1-Fc 293T Cells; # AMS. RSPO1-CELLS, RRID:CVCL_RU08, AMS Biotechnology, Abington, United Kingdom) with 10% BSA, containing factors critical for stem cell growth (Jung et al., 2011), including Recombinant Human Noggin ((0.1μgml^−1^,# (#120–10c, PeproTech, Rocky Hill, NJ, United States), Nicotinamide (1221.2μgml^−1^, #N3376–100G, MerckMillipore, Burlington, MA, United States), N-Acetyl-L-cysteine (163.2μgml^−1^, #A9165–25G, Sigma-Aldrich, MO, United States), Inhibitor of TGFβ type 1 activating receptor-like kinase (ALK5) A-83–01 (0.211μgml^−1^, #SML0788, Sigma-Aldrich), MAPK inhibitor SB202190 (3.31μgml^−1^, #S7067, Sigma-Aldrich), Human EGF (0.05μgml^−1^, #AF-100–15, PeproTech) and Gastrin (0.02μgml^−1^, G9145-.1MG, Sigma-Aldrich). For establishment of colonoids, the growth medium was supplemented with GSK inhibitor CHIRR99021 (1.16μgml^−1^, #72052, STEMCELL Technologies, Vancouver, Canada) and ROCK inhibitor Thiazovivin (0.78μgml^−1^, #72252, STEMCELL Technologies), while after passaging (every 7–10 days) the selective ROCK-inhibitor Y-27632 (3.203μgml^−1^, #1254, Bio-Techne, Minneapolis, MN, United States) was added during the first three days of culture. For passaging, colonoids were dissociated into single cell suspension by incubation with TrypLE Express (#12605028, Gibco™, Thermo Fischer Scientific) at 37°C for 10–15min, followed by gentle pipetting using a syringe with an 18G fill needle and centrifugation for 5min at 500 x g. The cells were then resuspended in Matrigel. For experiments, colonoids were cultured in 20% O_2_ and 5% CO_2_ at 37°C for 10 days, with medium change every 2–3 days, followed by four days of differentiation before experiments. In differentiation medium, the amount of Wnt-3A conditioned medium was reduced to 5% of the volume and Nicotinamide and SB202190 removed, while the pan-NOTCH inhibitor DAPT (4.3μgml^−1^, #2634, Bio-Techne) was added. During proinflammatory treatment experiments, A-83–01 was removed. For assays in low oxygen, half of the culture plates were moved from 20% O_2_/5% CO_2_ to a separate incubator (New Brunswick Galaxy 170R CO_2,_ Eppendorf) in which an additional N_2_ gas input was used to calibrate the oxygen level to 2% O_2_. The colonoids were kept at 2% O_2_/5% CO_2_ at 37°C for 40h before sample collection.

### Functional Assays in Human Colonoids

Fully formed, undifferentiated colonoids were passaged and seeded at 500,000 cells^.^ ml^−1^. Matrigel per well, determined by cell counting on an automated cell counter (Countess II Automated cell counter, Thermo Fischer Scientific). After differentiation as described above, proinflammatory treatment experiments were carried out using TNF (100ngml^−1^, #300-01A, PeproTech) and IL17 (100ngml^−1^, #200-17, PeproTech) diluted in growth medium, alone or in combination for 24h, with untreated colonoids receiving growth medium as controls, at 20% or 2% O_2_. For experiments at 2% O_2_, colonoids were pre-equilibrated for 16h before treatment and kept at 2% O_2_ until the assay was completed. At 16h, ligand preparations or growth medium without ligand (untreated) was added in 1/10 of the total volume of growth medium, to avoid complete reoxygenation in the 2% O_2_ conditions. Conditioned media were collected, centrifuged to remove cell debris, and stored at -80°C. Colonoids were collected using Cell Recovery Solution (CRS) (#734-0107, Corning, NY, United States) which depolymerizes Matrigel, and washed with PBS/0.1% BSA and centrifuged (500 x g) three times. Colonoid pellets were snap-frozen in liquid nitrogen and stored at -80°C or prepared for immunohistochemistry or protein measurement in cell lysate, as described below.

### Confocal Imaging of Colonoids

To assess morphology and presence of dead cells in the colonoid culture, high resolution imaging using a Leica TCS SP8 confocal microscope, equipped with an HC PL APO CS2 20x/0.75 NA water immersion objective (Leica Microsystems, Mannheim, Germany) was performed. Cells were stained with NucBlue^®^ Live reagent (Hoechst 33342) for all cell nuclei and propidium iodide for dead cell nuclei (ReadyProbes Viability Imaging kit, Blue/Red, #R37610, Invitrogen) added directly to the cell culture 10min prior to imaging. The samples were excited by the 405nm, and 561nm laser lines from a White Light Laser (WLL) and fluorescence emission detected sequentially by hybrid detectors (HyD) in the spectral ranges 420–500nm and 580–670nm, respectively. Images were captured using LAS X (RRID:SCR_013673, Leica) software, at 12-bit depth, with a pinhole corresponding to 1 airy unit (at 580nm), and were post processed by linear histogram stretch (normalization) and maximum Z-projections in Fiji (RRID:SCR_002285) (Schindelin et al., 2012). All confocal images are maximum Z-projections based on 40–50 optical sections.

### Flow Cytometry

Differentiated organoids were either maintained in 20% or transferred to 2% O_2_ for the last 40h before being harvested and dissociated into single cell suspension by incubation with TrypLE Express (#12605028, Gibco™, Thermo Fischer Scientific) for 20min and careful pipetting with a 1000µl tip. Cells were washed with PBS with 2% FCS and centrifuged at 240 x g, and then incubated for 15min at 4°C with Fc-blocking reagent and antibodies against CD326 (1:200) (#324233, APC-Fire, RRID:AB_2629702), CD24 (1:200) (#311123, BV605, RRID:AB_2562287), CD44 (1:200) (#103017, AF647, RRID:AB_493680), CD117 (1:200) (#313211, PE-CY7, RRID:AB_893228) all from BioLegend (San Diego, CA, United Staes). Further, rhodamine-labelled UEA1 lectin (1:500) (#RL-1062 1mgml^−1^, rhodamine, RRID:AB_2336769) from Vector laboratories (Burlingame, CA, United States) was used for identifying secretory cells. Antibodies and UEA-1 were diluted in PBS with 2% FCS. Samples were washed and stained with DAPI (1:1000 in PBS with 2% FCS) for live-dead exclusion, filtered through a 100µM mesh and immediately analyzed on a BD LSRII flow cytometer. BD CompBead anti-mouse Ig/anti-rat IG (#552843, RRID:AB_10051478, BD Biosciences, Franklin Lakes, NJ, United States), were used for antibody compensation controls. Post-acquisition analysis was carried out with FlowJo (RRID:SCR_008520, version 10).

### Immunohistochemistry of Colonoids

Colonoid-pellets were resuspended in 50µl Richard-Allan Scientific™ HistoGel™ Specimen Processing Gel (# HG-4000-012, Thermo Fisher Scientific) before being fixed in formalin for 24–48h and embedded in paraffin. Colonic biopsies were fixed in 10% buffered-formalin for 3–6days before embedding in paraffin. Formalin-fixed paraffin-embedded (FFPE) sections were processed through standard de-paraffinization and quenching of endogenous peroxidase before IHC. Antigen retrieval was achieved by 15min boiling in citrate buffer (pH 6.0) using a commercial microwave oven. All sections were incubated overnight at 4°C with primary mouse monoclonal anti-human antibody against HIF1α (#MA1-16504, RRID:AB_568567, Invitrogen, Thermo Fisher Scientific, MA, United States) or Ki67 (#M7240, RRID:AB_2142367, Dako Agilent, Santa Clara, CA, United States) diluted 1:20 (a-HIF1α) or 1:100 (a-Ki67) in PBS with 0.25% Triton-X and 0.25% BSA freshly used from frozen aliquotes. For a-HIF1α an additional 15min incubation with EnVision FLEX + Mouse (LINKER) (#K8021, Dako Agilent) was used. Immunoreactions were visualized using the rabbit/mouse EnVision-HRP/DAB + kit (#K5007, Dako Agilent) and counterstaining with hematoxylin. Negative controls were omission of primary antibody and similar concentration of matching-isotype non-immunized IgG. Images were captured by Nikon Eclipse Ci microscope, DS-Fi1 camera and NIS-Elements BR imaging software (Nikon Corporation, Tokyo, Japan). Further processing was done using Photoshop (Adobe Photoshop CC, 20.0.6 Release) and Fiji (RRID:SCR_002285)(Schindelin et al., 2012). Quantification of the Ki67 and HIF1α IHC staining was done using Fiji images with 10x and 40x magnification, respectively. In each group, a total number of 500-5000 (Ki67) or 250-800 (HIF1α) nuclei from several images were counted in four to five independent experiments, and the percentage of positive nuclei averaged within each experiment.

### Detection of Apoptosis in Colonoids

To assess apoptosis, the Caspase-Glo 3/7 Assay (#G8090, Promega, Madison, WI, United States) was used according to manufacturer’s instructions. The assay was performed three times, with three technical replicates, on colonoids grown in 96-well plates untreated or treated with ligands at 20% or 2% O_2_. Luminescence was measured on a Victor3 plate reader (PerkinElmer, Waltham, MA, United States).

### Blood Gas Analyzes of Conditioned Medium From Colonoids

An ABL90FLEX plus blood gas analyzer (Radiometer Medical, Brønshøj, Denmark) was used according to manufacturer’s instructions to measure and compare pH and concentrations of glucose and lactate in conditioned medium from colonoids grown at 20% or 2% O_2_ for the last 40h.

### RNA Extraction and Gene Expression Analysis

RNeasy Mini kit (#74106, Qiagen, Hilden, Germany) (colonoids) and RNeasy Micro Plus (#74034, Qiagen) (microdissected epithelial monolayer) were used for RNA extraction, according to the manufacturer’s protocol. For RNA-Seq of colonoids, the total RNA concentration was measured using a Qubit RNA HS Assay Kit on a Qubit 2.0 Fluorometer (Thermo Fisher Scientific, Waltham, MA, United States). Integrity was assessed using an Agilent RNA 6000 Nano Kit on a 2100 Bioanalyzer instrument (Agilent Technologies, Santa Clara, CA, United States). RNA sequencing libraries were generated using SENSE FFPE total RNA-Seq library prep kit (with RiboCop rRNA depletion) according to manufacturer’s instructions (Lexogen GmbH, Vienna, Austria). Libraries were normalized and pooled to 2.4nM and subject to clustering by a cBot Cluster Generation System on HiSeq4000 flowcells (Illumina Inc., San Diego, CA, United States), according to manufacturer’s instructions. The sequencing (75 cycles single end reads) was performed on an Illumina HiSeq4000 instrument, in accordance with the manufacturer’s instructions (Illumina). FASTQ files were created with bcl2fastq 2.18 (RRID:SCR_015058, Illumina). Transcript expression values were generated by quasi alignment using Salmon (Patro et al., 2017) and the Ensembl (GRCh38) human transcriptome. Aggregation of transcript to gene expression was performed using tximport (RRID:SCR_016752) (Soneson et al., 2015). Gene expression values with CPM (counts per million) below one in more than three samples were filtered out before differential expression analysis.

Laser capture microdissection (LCM) of epithelial cells from colonic biopsies was performed as previously described (Ostvik et al., 2020). Approximately 10,000 epithelial cells (1mm^2^) were acquired by LCM and cells were immediately lysed in RNeasy Micro Plus lysis buffer (300µl) and kept at -80°C. Illumina TruSeq RNA access library kit (Illumina) was used to prepare libraries for RNA-Seq, according to the manufacturer’s protocol.

### Quantitative Real-Time PCR

Total RNA isolated from colonoid pellets was stored in cryotubes in liquid nitrogen. The High-Capacity RNA-to-cDNA kit (Applied biosystems) was used for reverse transcription, and qPCR performed using PerfeCTa assay with PerfeCTa qPCR FastMix (Quantabio, Beverly, MA, United States) and Taqman probes (Applied biosystems), EPAS1: probe ID Hs01026149, CA9: probe ID Hs00154208, VEGFA: probe ID Hs00900054) with housekeeping genes (TBP: probe ID Hs0042760, ACTB: probe ID Hs01060665). StepOnePlus Real-Time PCR system with StepOne software version X (RRID:SCR_014281, Applied Biosystems) was used. Fold changes were calculated using the delta-delta CT method. RNA-seq data is available at Gene Expression Omnibus (GEO) under the accession number GSE172404.

### Bioinformatics and Gene Set Enrichment Analysis

Initial interpretation of data was done using the unsupervised dimensionality-reduction method of Principal Component Analysis (PCA). Differential gene expression of microdissected colonic epithelium were identified using R package limma (voom, RRID:SCR_010943) with an adjusted *p* value < 0.05 between paired experimental conditions. Differential gene expression of colonoids were identified using R package DESeq2 (RRID:SCR_015687) (Love et al., 2014) with an adjusted *p* value <0.05 between paired and unpaired experimental conditions. Enrichment analyses were done in MetaCore™ version 6.34 build 69,200 (Clarivate Analytics, Philadelphia, PA).

### Multiplex Chemokine Profiling and ELISA

The Bio-Plex Pro Human Chemokine Panel, 40-plex (#171ak99mr2, Bio-Rad laboratories, Hercules, CA, United States) was used according to the manufacturer’s instructions to detect cytokines in conditioned medium from colonoids stimulated at 20% or 2% O_2_. Of the 40 cytokines, 30 were chemokines and 10 belonged to other cytokine groups including interferons, IL-1 family and growth factors. Cytokines with release <100pg ml^−1^ were excluded from further assessment. ELISA kits for human CXCL1 (DY275-05), CXCL2 (DY276-05), CXCL5 (DY254-05), CXCL8 (DY208), CXCL10 (DY266-05), CXCL11 (DY672) and CCL20 (DY360), all from R&D Systems (Abington, United Kingdom), were used according to manufacturer’s protocols.

### Materials

Supplementary file SF2 lists materials described in the text above.

### Statistical Analysis

Except for the Bioinformatics and Gene set enrichment analyses described above, statistical analyses were performed in GraphPad Prism 8.0 (RRID:SCR_002798, GraphPad Software Inc., San Diego, CA, United States). For normally distributed numerical values and log2 transformed data, differences between groups were evaluated by one sample *t*-test, paired t-tests or ANOVA with multiple comparison tests with *p*-value correction, as indicated in the figure legends. Briefly, Tukey’s multiple comparison test was used when comparing every treatment with every other treatment and Holm-Šídák test when selected groups were compared. For data not following Gaussian distribution according to Shapiro-Wilk test, Kruskal Wallis test followed by Dunn’s multiple comparisons test were used to compare three or more groups, or Wilcoxon matched-pairs signed rank tests or Mann-Whitney U test were used to compare two groups. When ANOVA was used, post-hoc tests were conducted only if F was signi?cant and there was no variance in homogeneity. *p* < 0.05 was considered significant for all analyses. Data used for plotting and statistics are shown in Supplementary Data Sheet 2.

## Results

### Human Colonoids Were Viable and Responsive to IBD-Relevant Cytokines at 2% Oxygen

**FIGURE 1:**
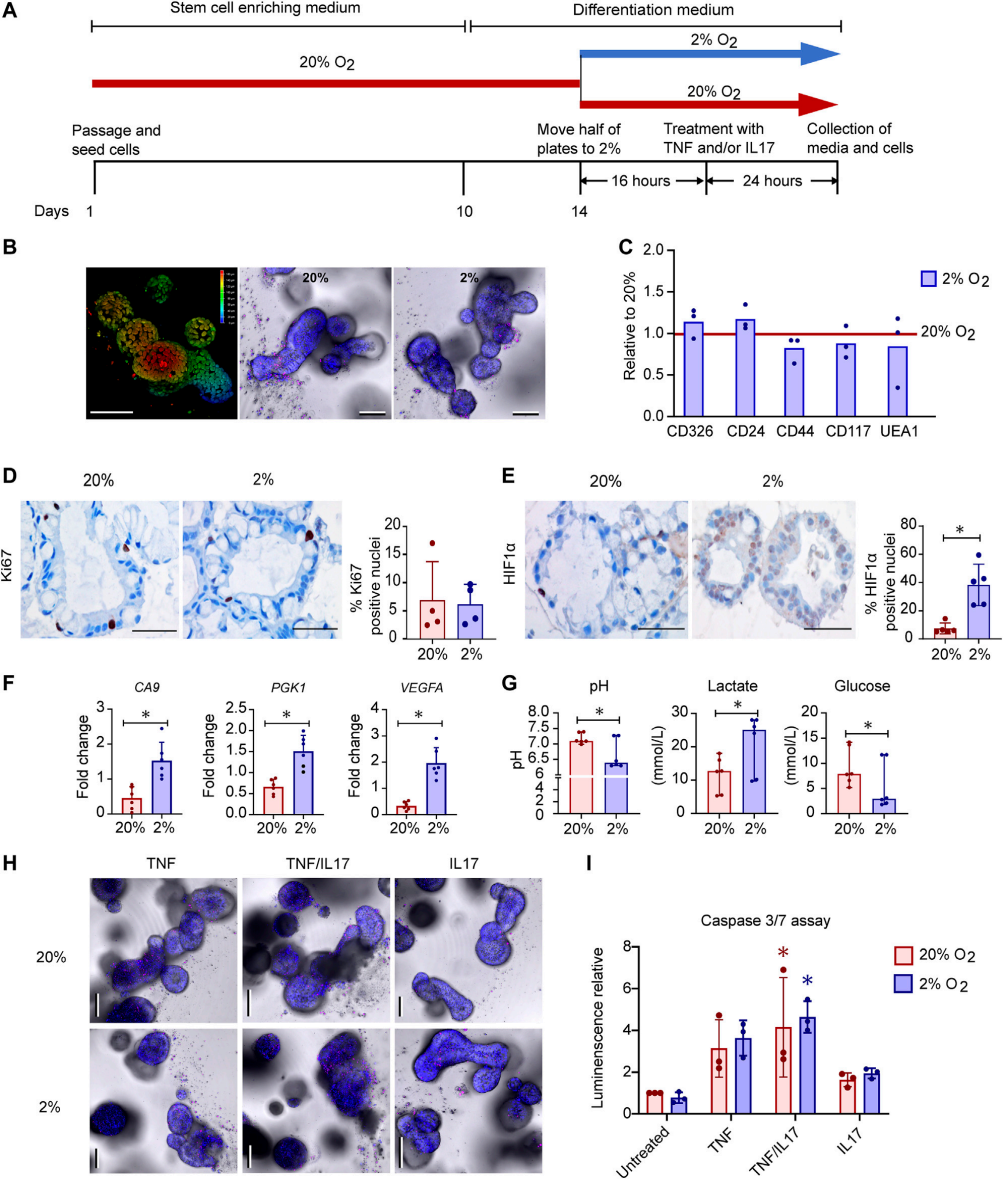
Effects of oxygen concentration on growth, cell-death, gene expression and metabolism in human colonoids **(A)**. Experimental design. Differentiated colonoids from six donors were either grown continuously at 20% O_2_ or calibrated at 2% O_2_ for 16h before treated with TNF (100ngml^−1^), IL17 (100ngml^−1^) or a combination (TNF/IL17) for 24h **(B)** Detection of morphology and cell death by live imaging of untreated colonoids. Left image shows a 3D depth encoded image of one colonoid at 20% O_2_, according to the color scale from red (superficial) to blue (deep) on the right side of the image. Center and right image panels show colonoids in 20% or 2% O_2_ respectively, where NucBlue^®^ Live reagent (Hoechst 33342) stains all cell nuclei (blue) and propidium iodide stains dead cell nuclei (red). Scale bars 100µm **(C)** Expression of epithelial cell markers as measured by flow cytometry in untreated colonoids from three donors, in 20% O_2_ or 2% O_2_ as depicted in panel **(A)** (*n* = 3 + 3 for each marker). Relative change between oxygen concentrations normalized to 20% O_2_ (red line at y = 1) displayed, with mean and individual normalized values **(D)** and **(E)** Immunostaining of differentiated colonoids grown at 20% or 2% O_2_, representative selection from five assays. Scale bars 50µm. **(D)** Ki67 positive nuclei stained brown, counterstaining with hematoxylin. The graph shows percentage of Ki67 positive nuclei in four independent experiments indicated by dots. Bars show mean with SD **(E)** HIF1α translocated to nuclei shown in brown counterstaining with hematoxylin. The graph shows percentage of HIF1α positive nuclei in five independent experiments indicated by dots. Bars show mean with SD. *p < 0.05 (Mann-Whitney U test) **(F)** Expression of the HIF target genes *CA9*, *PGK1* and *VEGFA* in untreated colonoids (*n* = 6) at 20% or 2% O_2_ (*n* = 6) by RT-qPCR. Mean fold change relative to housekeeping gene (*ACTB*) with SD and individual values is given. Paired t-tests were used to detect difference between 20 and 2% O_2_ (*p < 0.05) **(G)** pH, Lactate (mmol^.^L^−1^) and glucose (mmol^.^L^−1^) in conditioned medium from untreated colonoids (*n* = 6) grown at 20% or 2% O_2_ for 40h. Median with CI and individual values shown. Differences determined using Wilcoxon matched-pairs signed rank test (*p < 0.05) **(H)** Imaging of colonoids at 20% or 2% O_2_, after treatment for 24h with TNF or IL17 alone or in combination (TNF/IL17). Staining performed as in **(B)**. Scale bars 100µm **(I)** caspase 3/7 activity in untreated, TNF, TNF/IL17 or IL17 treated colonoids grown at 20% or 2% O_2_. Mean luminescence normalized to untreated at 20% O_2_ and individual values shown from three independent assays. Significant cytokine induced apoptosis determined by Kruskal Wallis test followed by Dunn's multiple comparison test (*p < 0.05). No difference found between oxygen concentrations (paired *t*-test).

Intestinal epithelial organoids are usually grown in 20% O_2_, which is much higher than available oxygen for intestinal epithelial cells *in vivo*. We hypothesized that lowering the incubator oxygen level from 20% to 2% would mimic *in vivo* physioxia in the colonoids and induce alterations relevant for IEC functions. Colonoids from 6 donors (Supplementary File SF1) were grown and differentiated into 3D structures with budding at 20% O_2_ (Figures 1A,B). Further, the colonoids were either transferred to 2% O_2_ or kept at 20% O_2_ for 40h prior to collection of media and cells for downstream assays (Figure 1A). Live imaging of colonoids showed that these were morphologically similar in 20% O_2_ and 2% O_2_ (Figure 1B).

To see whether reduced oxygenation induced changes in cell composition, colonoids grown at 20% or 2% O_2_ were dispersed into single cells and stained with the markers CD326 (expressed by colonic epithelial cells), CD24 (expressed by *e.g.*, stem cells), CD44 (expressed by *e.g.*, proliferating cells), CD117 and UEA1 (expressed by *e.g.*, secretory cells) (Figure 1C). We found no significant changes in expression of these markers with different oxygen concentrations. Moreover, IHC staining for Ki67 in sections of paraffin embedded colonoids did not show different proliferation rates between 20% and 2% O_2_ (Figure 1D).

Since hypoxia inducible transcription factors (HIFs) are stabilized and translocated to the cell nucleus in response to reduced oxygenation (Van Welden et al., 2017; Watts and Walmsley, 2019) we examined intracellular HIF1α expression in colonoids by IHC staining. HIF1α was detected in both cytoplasm and nuclei. Quantitative measurements showed significantly higher percentages of HIF1α positive nuclei in colonoids exposed to 2% O_2_ compared to 20% O_2_ (Figure 1E, Supplementary Data Sheet 2), indicating a nuclear translocation of the protein. Consequently, RT-qPCR showed that the expression levels of the classical HIF target genes carbonic anhydrase 9 (*CA9*), phosphoglycerate kinase 1 (*PGK1*) and *VEGFA* increased significantly in 2% compared to 20% O_2_ (Figure 1F). Blood gas analyses of conditioned medium from the colonoids showed that reduced oxygen concentration led to increased glucose consumption followed by lactate production, resulting in a decreased medium pH (Figure 1G).

Next, we examined how IBD-drug targets such as TNF, IL17 or a combination of both (TNF/IL17) altered colonoid morphology at different O_2_ concentrations. Live imaging with staining of dead cells and caspase 3/7 assay, showed a similar trend in both 20% and 2% O_2_, where TNF affected cell survival compared to untreated colonoids. Adding IL17 together with TNF enhanced cell death, while IL17 alone had no effect on morphology (Figure 1H) or caspase 3/7 activity (Figure 1I) when compared to untreated colonoids.

In sum, we induced a HIF response in colonoids when reducing oxygen level from 20% to 2% O_2_ for 40h, with upregulation of classical HIF target genes and a clear shift in metabolism as expected (Lanis et al., 2017). Moreover, the colonoids were responsive to TNF and IL17 in both 20% and 2% O_2_. The proinflammatory cytokine TNF induced, particularly in the combination with IL17, high level of cell death which was maintained across different O_2_ concentrations. Thus, the colonoids were fully viable at the 2% O_2_ level and exhibited unaltered morphology between 20% and 2% O_2_ when treated with IBD drug targets. This shows that colonoid assays can be performed in 2% O_2_ which may recapitulate better the *in vivo* physiological environment of colonic epithelial cells.

### Inflammation-Associated Genes Induced by TNF and TNF/IL17 Were Attenuated at 2% Oxygen

**FIGURE 2:**
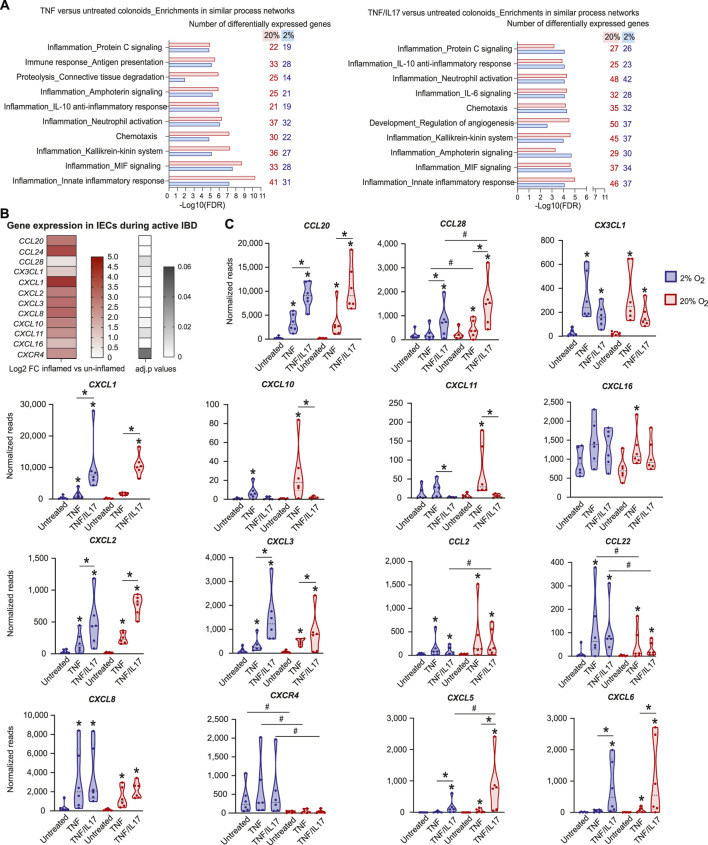
Effects of TNF and IL17 on inflammation-related genes in colonoids at 20% or 2% O_2_ **(A)** Enrichment analysis of RNA-Seq data from colonoids after treatment with TNF or TNF/IL17 compared to untreated control at 20% or 2% O_2_. Gene ontology (GO) networks strongly associated with the differentially regulated genes (adjusted *p* values are all <0.05) are shown, with bars illustrating the statistical significance of the association (−Log_10_ false discovery rate (FDR)). The numbers of genes associated with the GO networks at 20% or 2% O_2_ are listed on the right. Enrichment analyses were performed using MetaCore™ version 6.34 build 69200 (Supplementary Data Sheet 2) **(B)** Upregulated chemokine and chemokine receptor genes in microdissected colonic epithelium from active IBD (*n* = 12) compared to uninflamed epithelium (healthy controls and IBD in remission, *n* = 17) shown as Log_2_ fold change (heatmap with upregulated genes) and adjusted *p* values (gray scale) **(C)** Chemokine gene expression in colonoids from six donors, treated with TNF or TNF/IL17 for 24h in 2 and 20% O_2_. Examples of significantly (*p* < 0.05) regulated chemokines and receptors are shown, determined using LIMMA linear models with least squares regression and empirical Bayes moderated *t*-statistics with Benjamini Hochberg false discovery rate correction for multiple comparisons **(B,C)**. * without lines show significant alterations due to drug-target treatments when compared to untreated controls whereas * above lines show significant comparisons made between TNF and TNF/IL17. # above lines show significant differences between 2% oxygen and 20% O_2_ within a specific treatment condition.

In the complex cytokine landscape of both the healthy and the diseased gastrointestinal mucosa, TNF is an important IBD-associated proinflammatory cytokine (Murch et al., 1993; Breese et al., 1994; Nanki et al., 2020), and IL17 modulates the effect of other cytokines (Amatya et al., 2017). We therefore analysed the effects of TNF and TNF/IL17 on gene expression in colonoids grown under standard cell culture oxygen concentration of 20% and lower, more physiological level of 2%. There was no significant change in the expression of TNF-receptors *TNFRSF1A/1B* and the IL17 receptor *IL17RA* at 2% and 20% O2, or in untreated and cytokine treated colonoids (Supplementary Figure S1). Compared to untreated colonoids, cytokines regulated fewer genes in 2% O_2_ than in 20% O_2_ (Table 1, GEO, GSE172404). Enrichment analysis of gene expression in colonoids treated with TNF compared to untreated colonoids in 2% and 20% O_2_ showed a strong overrepresentation of genes in networks related to inflammation (Figure 2A, Supplementary Data Sheet 2) such as "Immune response_Antigen presentation”, “Inflammation_Neutrophil activation,” “Inflammation_IL-10 anti-inflammatory response,” “Inflammation_Innate inflammatory response,” and “Chemotaxis”. There was substantial overlap between top ten networks in the enrichment analysis for TNF or TNF/IL17 compared to the untreated colonoids. Overall, 20% O_2_ gave stronger associations to the network categories involved in inflammation compared to 2% O_2_, with more genes mapping to the networks, and this effect was most prominent for the networks “Chemotaxis” and “Inflammation_Innate inflammatory response.”

**TABLE 1:** Cytokine regulated genes in colonoids.

Contrasts	2% O_2_		20% O_2_	
	Upregulated	Downregulated	Upregulated	Downregulated
TNF vs untreated	391	304	467	380
TNF/IL17 vs untreated	574	782	757	925

Number of significantly regulated protein coding genes (adjusted *p*-value < 0.05) in TNF and TNF/IL17 treated colonoids compared to untreated controls (see GEO, GSE172404 for complete list of gene expression data).

### Colonoid Chemokine Responses to TNF and TNF/IL17 Showed Similarities to Inflamed Epithelium

In the intestinal mucosa, chemotaxis of immune cells is regulated by chemokines released from both immune cells and IECs (Skovdahl et al., 2015; Kvedaraite et al., 2016; Trivedi and Adams, 2018). Because chemokine release is one of the important immunoregulatory traits of the epithelium that may be fine-tuned by IBD-drugs (Trivedi and Adams, 2018) we first used RNA-Seq to identify the expression of chemokines in microdissected epithelium from IBD patients with active inflammation (*n* = 12), compared to non-inflamed mucosa from IBD patients in remission (*n* = 11) and healthy controls (*n* = 6). We found differential expression of several important chemokines and chemokine receptor (Figure 2B). To further investigate the regulation of epithelial chemokine expression, we compared chemokine and chemokine receptor gene expression in microdissected epithelium (Figure 2B) to the expression in colonoids (Figure 2C) at both 20% and 2% O_2_. The treatment with TNF or TNF/IL17 in colonoids led to altered/increased expression of the chemokines *CCL20*, *CCL28*, *CX3CL1*, *CXCL1*, *CXCL2*, *CXCL3*, *CXCL8*, *CXCL10*, *CXCL11* and *CXCL16* and the chemokine receptor *CXCR4,* which corroborated the chemokine expression in the microdissected epithelium from inflamed biopsies (Figure 2C, GEO, GSE172404). These data suggest that the colonoid model could be used to study chemokines that are overexpressed in IEC during active IBD.

The expression of chemokines *CCL20*, *CCL28, CXCL1, CXCL2, CXCL3 CXCL5* and *CXCL6* were more regulated by the TNF/IL17 treatment when compared to TNF treatment alone whereas the expression of *CX3CL1*, *CXCL10*, *CXCL11, CXCL16* and *CCL2* was driven more by TNF treatment in comparison to TNF/IL17 treatment. Moreover, similar levels of *CXCL8* and *CCL22* was observed across both TNF and TNF/IL17 treatment. Importantly, the RNA Seq analysis in colonoids also indicated that oxygen levels affected chemokine expression (Figure 2C, GEO, GSE172404). For example, the TNF or TNF/IL17 induced expression of *CCL28*, *CCL2*, *CXCL5*, *CXCL10*, *CXCL11* and *CXCL6* were, or trended towards being, downregulated at reduced oxygen concentration, while *CCL22*, *CXCL8* and receptor *CXCR4* expression were upregulated at 2% O_2_ compared to standard cell culture concentration.

**FIGURE 3:**
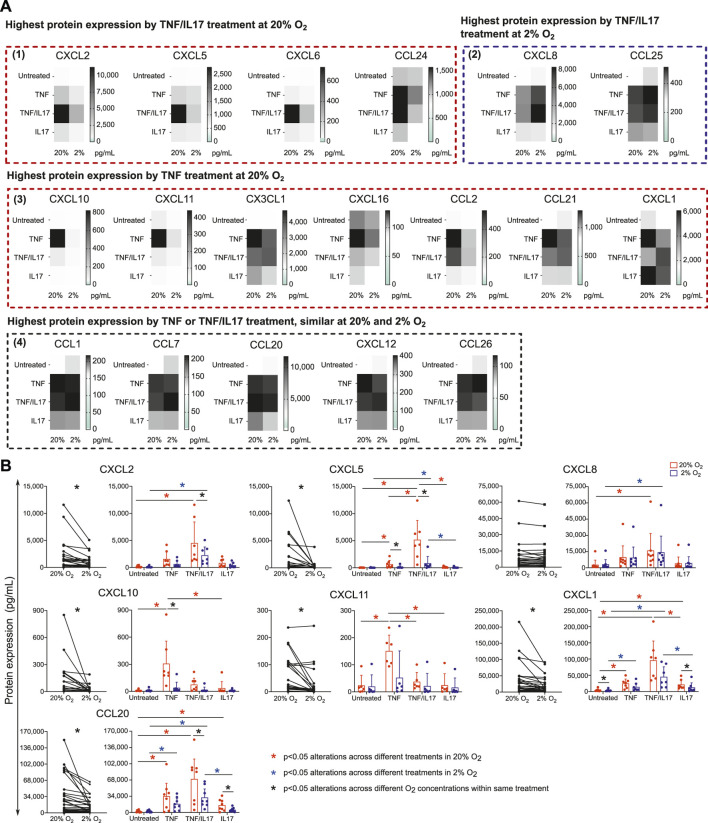
Chemokines released by colonoids at 20 or 2% O_2_. Conditioned medium from colonoids at 20 or 2% O_2_, followed by 24h treatment with TNF, IL17 or TNF/IL17, and untreated colonoids were analyzed for chemokine concentration **(A)** Mean protein concentration (*p*g^.^ml^−1^) of chemokines in conditioned medium from colonoids (*n* = 3 independent assays) analyzed by Human Chemokine Panel featuring 40 magnetic bead-based immunoassays. The 18 heatmaps show expression of significantly regulated proteins and are grouped according to 1) Highest protein expression by TNF/IL17 treatment at 20% O_2_. 2) Highest protein expression by TNF/IL17 treatment at 2% O_2_. 3) Highest protein expression by TNF treatment at 20% O_2_, and 4) Highest protein expression by TNF or TNF/IL17 treatment, similar at 20 and 2% O_2_ **(B)** CCL20, CXCL1, CXCL2, CXCL5, CXCL8, CXCL10 and CXCL11 protein concentrations (*p*g.ml^−1^) in conditioned medium from minimum six assays measured by ELISA. Left panels: paired data at 2 or 20% O_2_ across all treatments for the selected chemokines, analyzed with Wilcoxon matched-pairs signed rank test. Significance level is indicated as *p* values or not significant (ns). Right panels: concentrations for each chemokine in 2% (blue) or 20% (red) O_2_ plotted as individual values with mean and SD. Statistical analysis were performed on log2 transformed data (Supplementary Data Sheet 2). Alterations across different treatments within each oxygen concentrations were analyzed with one-way ANOVA followed by Tukey’s multiple comparisons or Kruskal-Wallis test followed by Dunn’s multiple comparisons test. Alterations across different oxygen concentrations within same treatment were analyzed by two-way ANOVA followed by Holm-Šídák multiple comparisons test. *p < 0.05; red and blue asterisks show comparisons across different treatments within 20 and 2% O_2_ conditions, respectively. Black asterisks indicate significant comparisons between 2 and 20% oxygen concentrations within a specific treatment.

We further examined alterations in chemokine protein release upon stimulation with IBD drug targets across different O_2_ concentrations. Conditioned medium from untreated colonoids and TNF or TNF/IL17 treated colonoids at 2% and 20% O_2_ were screened by a multiplex assay measuring 40 cytokines. We found that TNF or TNF/IL17 treatment induced release of 18 different chemokines into the conditioned media. (Figure 3A, Supplementary Data Sheet 2). These included nine of the chemokines upregulated in epithelium from IBD patients (Figure 2B, CCL20, CCL24, CX3CL1, CXCL1, CXCL10, CXCL11, CXCL16, CXCL2, CXCL8). The multiplex screening further suggested a reduced release of 11 chemokines in 2% O_2_ compared to 20% O_2_ (Figure 3A panels 1 and 3). Two chemokines had increased release in 2% O_2_ (panel 2) and five chemokines had similar release in both oxygen concentrations (panel 4).

To confirm multiplex results, we performed ELISA on conditioned medium from six donors (3 healthy controls + 3UC) for CXCL1, CXCL2, CXCL5, CXCL8, CXCL10, CXCL11 and CCL20 (Figure 3B, Supplementary Data Sheet 2), which verified epithelial release and an effect of TNF and/or TNF/IL17 treatment. Further, the release of all these chemokines except for CXCL8 was attenuated in 2% compared to 20% O_2_ and were in line with observations made with the multiplex data.

### Donor Differences in Cytokine Responses at 20% and 2% O_2_

**FIGURE 4:**
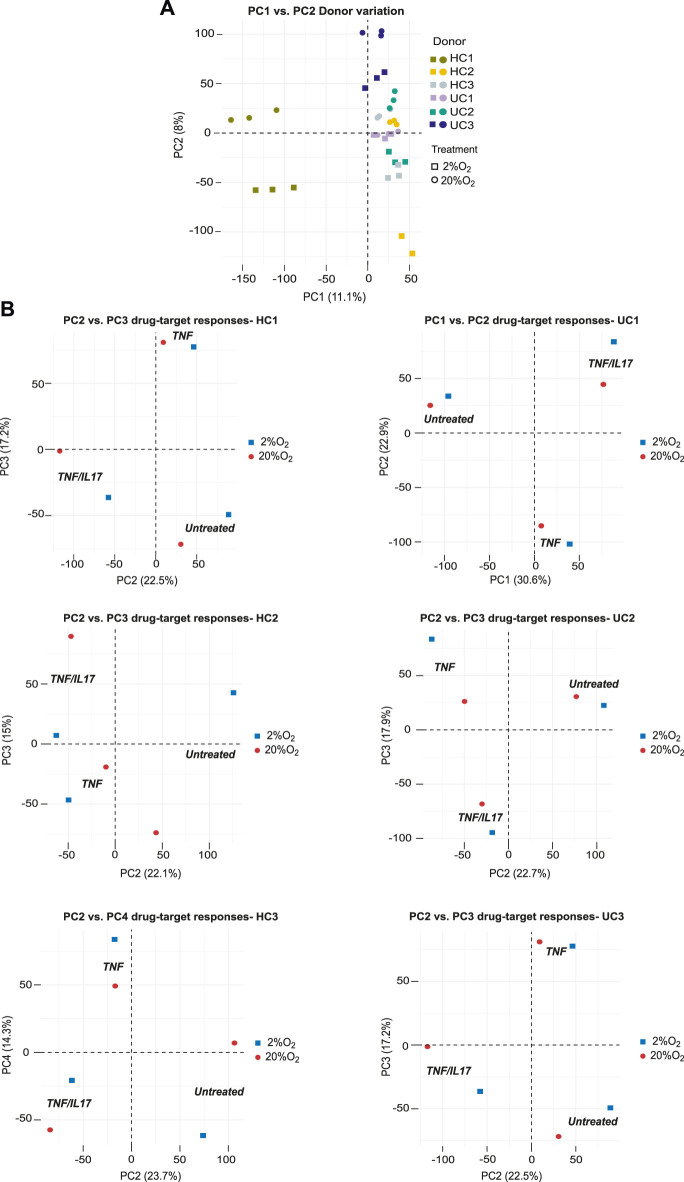
Donor differences in global gene expression. The panels show unsupervized analysis (PCA) of RNA-Seq data of colonoids from six donors grown continuously at 20% or at 2% O_2_ for the last 40h **(A)** PCA of the complete dataset, PC1 vs PC2 captures the influence of donor variations and oxygen level. The different donors and oxygen concentrations are indicated by color coded symbols. Three treatment conditions per donor in each oxygen concentration (*n* = 6) are shown **(B)** PCA of data from each donor. Plot of PC2 vs. PC3 captures the effect of treatments in donors HC1-2 and UC2-3, while PC2 vs. PC4 and PC1 vs PC2 captures this effect best for HC3 and UC1, respectively.

Since patient-derived organoids retain donor characteristics they may be used to study donor-differences in response to extracellular stimuli and drug-target-responses. Unsupervised analysis (PCA) of global gene expression across all conditions in colonoids from the six donors showed indeed that the dominating sources of variation in the two first principle components (19.1% explained variance) separated donors and oxygen levels into distinct clusters (Figure 4A). Inspecting the first four components in the PCA analyses of colonoids from the six donors individually, clear separations (22.1%–30.6% explained variance) were seen between untreated vs. TNF or TNF/IL17 treated colonoids. We also observed clear separation between TNF and TNF/IL17 treated colonoids as well as between oxygen levels in varying sets of the four dimensions PC1 to PC4 (Figure 4B). Thus, the drug-targets TNF/IL17 induced strong responses in all donors that also could be separated by oxygen level.

**FIGURE 5:**
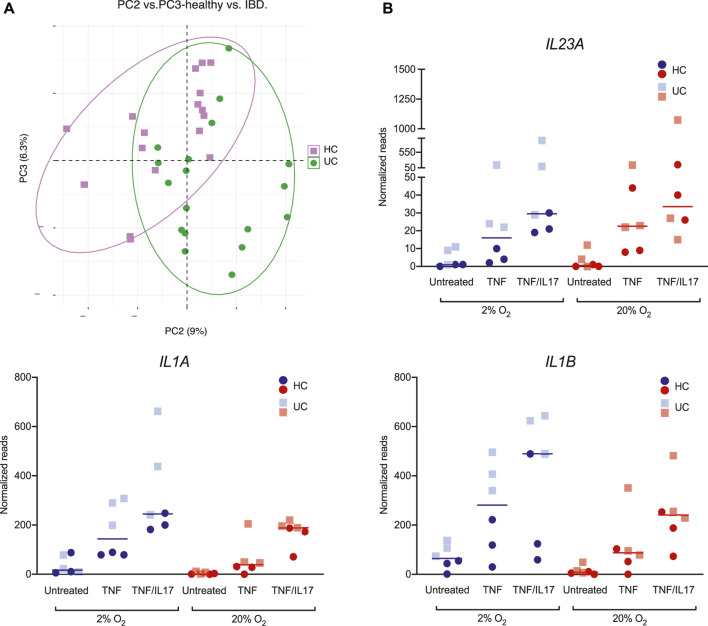
Donor differences in gene expression of *IL23*, *IL1Α* and *IL1Β* **(A)** PCA of colonoid gene expression in global gene expression data from ulcerative colitis (UC, green) and healthy controls (HC, purple). Plot of PC2 vs PC3 captures the group effect **(B)** Expression of the IBD-relevant cytokines *IL23A*, *IL1Α* and *IL1Β* in colonoids from HC (circles) and UC (squares), in 20% (red) or 2% (blue) O_2_ and treatment with TNF, TNF/IL17 or untreated control. Individual values (normalized reads) with median are shown.

In the PCA of global gene expression across all conditions in colonoids from the three HC donors and three UC donors, we also observed a moderate clustering based on disease status. Despite the individual donor variances, the second and third principle components captured the difference between HC and UC colonoids (untreated, TNF, TNF/IL17 in 2% and 20% O_2_) (Figure 5A). Exploring the gene lists (GEO, GSE172404) we found that *e.g.*, the highly relevant IBD-cytokines *IL23*, *IL1Α* and *IL1Β* were higher expressed in colonoids from UC compared to healthy control colonoids upon TNF and TNF/IL17 treatment, particularly in 2% O_2_ (Figure 5B). Thus, we found differential expression of genes relevant to mucosal inflammation between UC and healthy control donors upon treatment with proinflammatory triggers in 2% O_2_, which would not be detected in 20% O_2_.

## Discussion

Although it is well known that physical, biochemical and immunologic driven barrier dysfunctions of epithelial cells have critical roles in the pathogenesis and perpetuation of IBD (Kaser et al., 2010; Parikh et al., 2019), there is limited information about how drugs commonly used in IBD act on the epithelium in disease. Moreover, the response to a given drug can not be predicted on the individual level in daily clinical practice. To achieve individualized, targeted therapies in IBD, we need a better understanding of this clinical heterogeneity observed among patients. We have previously confirmed that organoids developed from human colonic tissue (colonoids) contain stem cells and all differentiated cell lineages present in the colonic epithelium, and develop a 3D structure with budding crypts and surface areas encircling the lumen (Ostvik et al., 2020). Thus, from our results and those from others (Dotti et al., 2017; Arnauts et al., 2020; Sayoc-Becerra et al., 2020) colonoids emerge as a putative tool to study effects of existing or emerging IBD treatments targeting epithelial cells even in a specific patient.

To best utilize an *in vitro* model experimental conditions should closely mimic the *in vivo* environment. This concept is used also in other fields of biology e.g. during culture of haematopoietic stem cells (Jez et al., 2015) where in particular oxygen concentration has been found to be important. We thus chose to study the effect of oxygen at a low level physiological for the colonic mucosa, and compare with the commonly used atmospheric oxygen concentration which leads to a cellular oxygen concentration never seen *in vivo* (Keeley and Mann, 2019; Okkelman et al., 2020). Our results from experiments at 2% O_2_ reflect the findings by others, where during low oxygen levels, the HIF1 complex translocates to the nucleus initiating a transcriptional program (Van Welden et al., 2017; Watts and Walmsley, 2019) aimed both at adjusting the cell metabolism to consume less oxygen, and to maintain cell integrity. Essential for the colonoid model, we found no differences in cell composition, morphology or cellular apoptosis suggesting that colonoids cultured under a physiological, low oxygen concentration were fully viable. Relevance to IBD is suggested by studies in experimental colitis models (Sun et al., 2019; Colgan et al., 2020; Yin et al., 2020), and cell lines (Muenchau et al., 2019; Vissenaekens et al., 2019), which indicate a protective stabilization of HIF1α, attenuating mucosal inflammation and promoting barrier formation. Moreover, reagents that activate HIF *via* inhibition of the prolyl hydroxylase enzymes, are suggested to induce hypoxia-mediated resolution in patients with intestinal mucosal inflammatory disease (Glover and Colgan, 2011; Brown et al., 2020).

Further experiments were done to compare immunoregulatory responses to the highly IBD-relevant proinflammatory cytokines TNF/IL17 at 20% and 2% O_2_. Our main finding was that 20% O_2_ gave stronger associations to inflammation-associated gene network compared to 2% O_2_. We continued to focus on chemokines since our previous studies (Ostvik et al., 2013; Skovdahl et al., 2015; Ostvik et al., 2020) and analysis of chemokine mRNA level in inflamed microdissected epithelium indicate that intestinal epithelial cells express and release several chemokines during active IBD that can affect the recruitment of both inflammatory and regulatory immune cells to the intestinal mucosa. A better understanding of how these chemokines are regulated is important for targeted therapy (Trivedi and Adams, 2018). Our data from human colonoids demonstrated that TNF/IL17 increased both expression and release of chemokines known to attract neutrophil granulocytes and monocytes (CXCL1, CXCL2, CXCL5, CXCL8), compared to TNF alone. A similar response *in vivo* may thus reinforce an acute response through neutrophil attraction. In support of this, previous studies on IL17 receptor deficient mice show reduced neutrophil recruitment and increased susceptibility to infections (Ye et al., 2001). TNF/IL17 treatment in colonoids induced mRNA expression and release of CCL20, whereas expression and release of CXCL10 and CXCL11 were reduced, compared to TNF alone. CCL20 is reported to attract effector Th17 cells, T-regulatory cells (Treg) and plasma cells (Williams, 2006; Wang et al., 2009). CXCL10 and CXCL11 are thought to have opposite roles, where CXCL10 preferentially attract inflammatory Th1 cells and NK cells, whereas CXCL11 attracts Treg1 cells (Karin and Razon, 2018). Thus, a combined effect of TNF/IL17 in intestinal epithelial cells *in vivo* and the subsequent release of CXCL10, CXCL11 and CCL20 may influence the balance between the effector and regulatory T-cells, with an influx of Th17 cells, as discussed by (Lee et al., 2008).

While TNF/IL17 induced inflammatory responses at both 20% and 2% O_2,_ the effect on *CXCL1*, *CXCL2*, *CXCL5*, *CCL20*, *CXCL10* and *CXCL11* mRNA expression and protein release was less prominent at 2% O_2_. The overall assessment of our data is a reduction in TNF and TNF/IL17 induced responses in 2% O_2_, corresponding to the anti-inflammatory traits of hypoxia seen in other experimental systems (Sun et al., 2019; Colgan et al., 2020; Yin et al., 2020). Thus, our results suggest that if correctly tuned, a HIF-response in the epithelium may partly normalize the chemokine levels seen in the inflamed mucosa. More important, our results indicate that functional studies performed in patient-derived colonoids at unphysiological oxygen level might exaggerate cellular responses to drug-targets and thereby mask more subtle differences. Although we found a considerable donor-dependent variance in the colonoid assays, we were able to detect substantial oxygen-dependent differences in gene expression in untreated as well as TNF/IL17 treated colonoids in all donors. Interestingly, for some of the IBD-relevant cytokines donor differences based on disease status were more pronounced in 2% O_2_ than 20% O_2_.

In the present study we show that human colonoids adapted well to short time low (2%) oxygen. The cultures were maintained at rather low colonoid density to minimize heterogeneity in oxygen level and nutrients in the wells. A limitation of the study is that we did not measure real time O_2_ concentration and dynamics in our cultures at 20% and 2% O_2_. Generally, 3D organoid cultures can have variable oxygen concentration due to the media height, oxygen diffusion within the Matrigel, individual organoid size, oxygen consumption rates and metabolic demands for different cell types (Okkelman et al., 2017; Okkelman et al., 2020). By live cell imaging of mouse small intestinal organoids (enterocytes) grown at 20% O_2_, Okkelman et al. (2017) showed that O_2_ diffusion provided rather uniform oxygenation in the Matrigel matrix. However, their method revealed pronounced heterogeneity in oxygenation (2.8–9.7% O_2_) both within and between individual organoids, that might be explained by differences in aerobic metabolism of various cell types. Studies in 2D cell line cultures indicated an optimal physioxic range low enough to reduce oxidative damage but high enough for efficient oxidative metabolism for different cell types. (Ferguson et al., 2018; Timpano et al., 2019). Finding an optimal oxygen concentration that mirrors the *in vivo* physiological range in colon (<1%–3%), might require some optimization depending on the type of experiments, organoid density and differentiation protocol used. Growing organoids in smaller droplets or reseeding to 2D monolayer could eliminate some variability in local O_2_ environment across a culture well.

Organoid models that mimic human physiology have potential to both reduce the reliance on animal models for drug-testing and improve development of personalized medicine in humans. To our knowledge this is the first study that examines the effects of the IBD drug-targets TNF/IL17 on colonoids at a more physiologically relevant oxygen concentration. Our findings in the colonoids strongly indicate that an oxygen concentration closer to the *in vivo* epithelial cell environment is of essence in IBD-related experimental pharmacology. Although our focus in the present work is in the context of IBD, intestinal organoids can be used for drug-testing and experimental pharmacology related to several other diseases, including cancer. Future studies should focus on fine-tuning the optimal oxygen conditions for long term as well as short term studies, and determine possible cellular differences in oxygen demands to enhance the translational value of the model and gain insight into intestinal pathobiology.

## Data Availability

The datasets presented in this study can be found in online repositories. The names of the repository/repositories and accession numbers can be found below: GEO, GSE172404.

## Abbreviations

FFPE, Formalin-fixed paraffin-embedded; HIF, hypoxia inducible factor; IHC, immunohistochemistry; IBD, inflammatory bowel disease; IL17, interleukin 17; IEC, intestinal epithelial cell; PGK1, phosphoglycerate kinase 1; PCA, principal component analysis; PHDs, prolyl hydroxylases; UC, ulcerative colitis; RNA-Seq, whole transcriptomic shotgun sequencing.
