# Supplementary material for: Patient Derived Colonoids as Drug Testing Platforms–Critical Importance of Oxygen Concentration
Source: Front Pharmacol. 2021 May 13;12:679741. doi: 10.3389/fphar.2021.679741 (PMC8156408; doi:10.3389/fphar.2021.679741)
Supplement: Supplementary file 1 [file Image1.pdf]

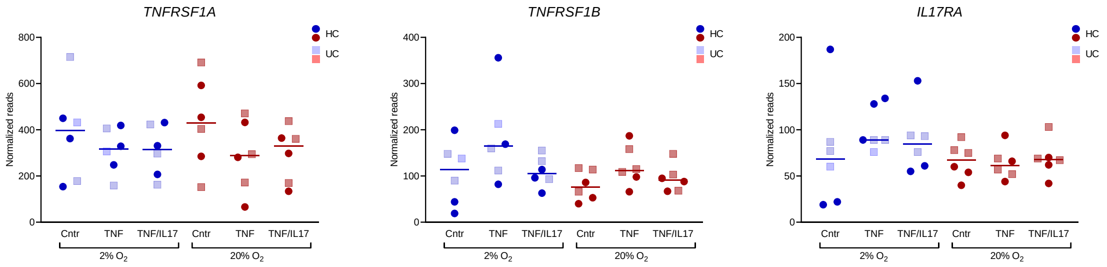

Supplementary Figure SF1: **Receptors for TNF and IL-17.** Expression of TNFRSF1A/1B and IL17RA in colonoids derived from healthy controls (circles) and patients with UC (squares) in 20% (red) or 2% (blue) O_2 and treatment with TNF, TNF/IL17 or untreated control. Individual values (normalized reads) with median are shown.
